# Maternal stress in pregnancy and adverse birth outcomes among women living with HIV during the COVID-19 pandemic

**DOI:** 10.3389/frph.2026.1900562

**Published:** 2026-08-25

**Authors:** Paige L. Williams, Jessica Lee, Deborah Kacanek, Ellen G. Chadwick, Haleigh Civiletto, Claire A. Berman, Ann Aschengrau, Liz Salomon, Sue Siminski, Kathleen M. Powis

**Affiliations:** 1 Department of Biostatistics, Harvard T.H. Chan School of Public Health, Boston, MA, United States; 2 Center for Biostatistics in AIDS Research, Harvard T.H. Chan School of Public Health, Boston, MA, United States; 3 Department of Epidemiology, Harvard T.H. Chan School of Public Health, Boston, MA, United States; 4 Department of Pediatrics, Ann & Robert H. Lurie Children’s Hospital of Chicago, Chicago IL, United States; 5 Frontier Science & Technology Research Foundation, Inc., Amherst, NY, United States; 6 Department of Epidemiology, Boston University School of Public Health, Boston, MA, United States; 7 Departments of Internal Medicine and Pediatrics, Massachusetts General Hospital, Boston, MA, United States; 8 Department of Immunology and Infectious Diseases, Harvard T.H. Chan School of Public Health, Boston, MA, United States

**Keywords:** COVID-19, discrimination, HIV, preterm, small for gestational age, stress, stressful life events

## Abstract

**Background:**

Stress, depression, and racial discrimination occurring in pregnant women have demonstrated associations with adverse birth outcomes, but their role during and after the COVID-19 pandemic among women living with HIV (WLHIV) has not been evaluated.

**Methods:**

We conducted surveys in pregnant and postpartum WLHIV participating in the US-based SMARTT study, asking them to report their experiences of social distancing, trauma and violence, and changes in housing, income, sleep and energy levels resulting from the COVID-19 pandemic. Women were also asked about their levels of perceived stress during pregnancy, experiences of discrimination, and depression and anxiety symptoms. Log-binomial regression models were used to evaluate associations of pandemic-related factors with perceived stress; linear and log-binomial models were used to evaluate associations of perceived stress with adverse birth outcomes.

**Results:**

Among 125 WLHIV, 36.0% reported stress during pregnancy, 12.2% had moderate/severe depression symptoms, and 17.5% reported high discrimination levels. In adjusted models, social distancing behavior, impact of the pandemic on daily life, change in housing status, intimate partner violence, poor sleep quality, lower energy levels, and moderate/severe depression symptoms were associated with two- to five-fold higher risk of perceived stress in pregnancy. However, no association was observed between perceived stress and adverse birth outcomes.

**Conclusions:**

Among WLHIV, over one-third experienced stress during pregnancy, associated with social distancing, trauma and violence, and other changes related to the COVID-19 pandemic. Optimal strategies are needed to screen pregnant and postpartum WLHIV for stress and mental health conditions and provide appropriate support during public health emergencies.

## Introduction

Stress, anxiety, depression, and exposure to structural racism and discrimination during pregnancy are associated with adverse outcomes for pregnant women and their infants, increasing the risk of maternal mortality, preterm birth, low infant birth weight, and neurodevelopmental deficits among children (1–5). Similarly, maternal stress, anxiety, and depression in the postpartum period have been associated with poor health outcomes for both women and their infants (6, 7). Preterm birth and small for gestational age (SGA) have been shown to contribute to both short- and long-term health and developmental problems for both mothers and their infants (8–15). Pregnant and postpartum women living with HIV (WLHIV)—an understudied group, with at least 3,500 pregnancies annually in the United States (U.S.)—may be particularly vulnerable to stress, given the added burden of managing HIV, preventing perinatal HIV transmission, and caring for their newborn (16).

Economic challenges and societal responses to the COVID-19 pandemic, including loss of income, housing instability, food insecurity, and social/physical distancing increased levels of stress, anxiety and depression in the U.S. (17–22). During the pandemic, 32% of U.S. adults reported symptoms of anxiety and depression, with higher levels among women than men (36% vs. 28%) (23). The pandemic was especially challenging for pregnant and postpartum women, partially due to disruptions in prenatal care and delivery practices (24, 25). In addition to fear of COVID-19 infection, pregnant women experienced anxiety around preparedness for the birth of their infant, particularly given maternity ward restrictions on visitors (26). Feelings of stress, anxiety, and depression often reported during pregnancy were heightened as a result of the pandemic (24, 27–30). Davenport and colleagues reported an increase in self-reported depression from 15% pre-pandemic to 41% during the pandemic, with even greater increases in anxiety (29% to 72%) (29). Similarly high levels of depression (47%), anxiety (60%) and stress related to the psychological impact of the pandemic (40%) were also reported among pregnant women in the UK (28).

Pregnant women in the US living with HIV, who predominantly identify as Black or Hispanic, likely experienced even greater increases in mental health conditions during the COVID-19 pandemic, given the healthcare inequities for minoritized populations (31–33). Severity of COVID-19 disease was reported to be higher among individuals identifying as Black or Hispanic (34–39). Racial and ethnic disparities exist in rates of preterm birth and low birth weight stemming in part from policies, institutional practices, and cultural norms that perpetuate structural racism (40–42). Given the syndemic of structural racism and the COVID-19 pandemic, examining the joint effects of stress experienced during the pandemic with adverse birth outcomes in pregnant WLHIV is particularly urgent (31, 39, 40).

The Surveillance Monitoring for ART Toxicities (SMARTT) Study conducted by the Pediatric HIV/AIDS Cohort Study (PHACS) network followed pregnant WLHIV and their children who are HIV-exposed and uninfected (HEU) at US-based sites between 2007 and 2025 (43–45). Over 65% of participants self-identified as Black or African American and nearly 30% self-identified as Hispanic. We conducted a substudy nested within SMARTT to estimate the prevalence of perceived stress, characterize specific types of Stressful Life Events, or “stressors”, experienced by pregnant WLHIV during the COVID-19 pandemic, and identify associations between perceived stress and birth outcomes. We hypothesized that pandemic-related challenges—social distancing, home schooling, intimate partner violence (IPV), depression, experiences of structural racism and discrimination, and economic strain—would increase stress among pregnant and postpartum WLHIV. We also hypothesized that increased stress levels would be associated with higher risk of preterm birth and SGA and lower infant anthropometrics at birth.

## Methods

### Study population

The SMARTT study was a prospective observational cohort study which opened in 2007 and enrolled WLHIV of reproductive age and their HEU infants at 22 clinical sites across 9 US states and Puerto Rico, as previously described (43–45). Women with HIV were enrolled during pregnancy or within 1 week after delivery; infants were enrolled prenatally or shortly after birth. Over 5,000 children born to WLHIV enrolled, along with over 3,800 mothers/caregivers (many of whom enrolled multiple children). Follow-up visits occurred annually through age 5 years and then biannually until age 17 years. The SMARTT Maternal Stress Study began in 2023 as a substudy nested within SMARTT Version 6 (V6) at 17 active SMARTT sites to evaluate stress and mental health of pregnant and postpartum women during and shortly after the COVID-19 pandemic; all women enrolled during pregnancy or attending their child's age 1 year study visit between July 2023 and December 2024 were automatically enrolled in the Maternal Stress Study upon consenting to SMARTT V6 study participation. The SMARTT V6 study, including the Maternal Stress Substudy, was approved by the Institutional Review Board (IRB) at Harvard T.H. Chan School of Public Health, which served as the single IRB of record; study sites ceded regulatory authority to the Harvard IRB. All participants provided written informed consent for their own participation and that of their infant.

### Outcomes and covariates

For the Maternal Stress Study, WLHIV completed an online “COVID-19 Impact Survey” either during pregnancy or at their child's age 1 year study visit (e.g., 1 year postpartum), to report on their experiences during and shortly after the COVID-19 pandemic. Slightly different surveys were created for pregnant vs. postpartum women, but both survey versions included a similarly-worded question regarding perceived stress during their pregnancy (“In general, what level of stress have you experienced during your pregnancy due to COVID-19?” for pregnant women; “How stressed did you feel during your pregnancy due to the pandemic?” for postpartum women) with the same outcome levels (“No stress”, “Slightly stressed”, “Moderately stressed”, or “Highly stressed”). This self-reported stress was considered the primary stress-related outcome. Participants also completed the Patient Health Questionnaire (PHQ-9) and Generalized Anxiety Disorder (GAD-7) assessments, which are validated screening tools for depression and anxiety symptoms, respectively (46, 47). We considered screening positive for depression based on moderate or severe symptoms (PHQ-9 score ≥ 10), and screening positive for anxiety based on moderate or severe symptoms (GAD-7 score  ≥ 10). Gestational age, birth weight, and birth length were abstracted from medical records, and used to classify preterm birth (<37 completed weeks gestation), SGA (birth weight <10th percentile for gestational age) and weight and length z-scores for gestational age based on CDC growth standards (48).

A separate validated online Stress and Discrimination Survey was completed by participants to collect information on occurrence of stressful life events and perceived discrimination. For pregnant women, stressors experienced were collected using the 13-item Pregnancy Risk Assessment Monitoring System-7 (PRAMS-7) Questionnaire developed by the Centers for Disease Control and Prevention (CDC) (49–51). For postpartum women, the Stress and Discrimination Survey included questions on occurrences of stressors from the Stressful Life Events (SLE) questionnaire, with 23 items (52). Although the individual questions differed between the PRAMS and SLE questionnaires, both included questions which could be grouped into domains of emotional, financial, partner-associated, and traumatic stressors (see Supplementary Table S1). Perceived discrimination was evaluated using the Everyday Discrimination Scale (EDS) (53).

The COVID-19 Impact Survey collected experiences of social distancing; trauma and violence; changes in housing, sleep, energy level and overall emotions; and changes in income and food insecurity during and shortly after the COVID-19 pandemic. Standardized instruments from the NIH PhenX Toolkit were used to collect data on trauma and violence at community and individual levels (COVID-19 Community Response Survey), intensity of emotions and feelings [Coronavirus Perinatal Experiences—Impact Survey (COPE-IS)], changes in housing and income (Housing and Income Survey), and food insecurity (U.S. Household Food Security Survey) (54). Engaging in social distancing behavior was defined as endorsing at least one of the individual social distancing behaviors. Intimate partner violence (IPV) was classified as a “yes” response to any individual question related to emotional, physical or sexual violence in the past year. Each of the changes in sleep, energy levels, or overall impact on daily life was considered as a separate predictor of stress. A decline in household income or change in housing were each considered as predictors.

In order to understand other potential predictors of perceived stress in addition to pandemic-related factors and mental health (depression and anxiety) during pregnancy, we also considered the following demographic and health-related covariates: age at delivery (years), race and ethnicity, U.S. region (based on participant's clinical site), education level, household income level, mode of HIV acquisition [perinatally acquired HIV (PHIV) or non-perinatally acquired HIV (NPHIV)], pre-pregnancy body mass index, (BMI, kg/m^2^), suppressed viral load (<50 copies/mL) early in pregnancy, COVID-19 infection during pregnancy, social support program assistance, substance use (tobacco, alcohol, or marijuana use during the first trimester), and antidepressant use during the first trimester.

### Statistical analysis

Participant characteristics were summarized by whether the participant was pregnant or postpartum (non-pregnant) at the time of completing the COVID-19 Impact Survey. Perceived stress level during pregnancy was summarized descriptively by the percent reporting each level of stress. For modeling purposes, perceived stress was dichotomized as any stress (slightly, moderately or highly stressed) vs. no stress. From the PRAMS-7 and SLE questionnaires, the number and percent of pregnant and non-pregnant participants who endorsed at least one stressor from each domain (emotional, financial, partner-associated, traumatic, and overall) were reported. The distribution of responses to each of the stressors were summarized by perceived stress level (“Not stressed”, “Slightly stressed”, or “Moderately/Highly stressed”) separately for pregnant and non-pregnant participants. Responses to individual questions on the COVID-19 Impact Survey related to social distancing; trauma and violence; housing, income and food insecurity; and changes in sleep, energy and overall emotions during the COVID-19 pandemic were also summarized for pregnant and postpartum women.

Separate log binomial regression models were fit to evaluate the association of each pandemic-related factor (social distancing; trauma and violence; changes in housing, sleep, energy level and overall emotions; and changes in income and food insecurity during the COVID-19 pandemic) with the risk of reporting any stress, yielding risk ratios (RRs) with 95% confidence intervals (CIs). Unadjusted and adjusted models were fit, with adjusted models including covariates selected as appropriate for that factor. All models were also adjusted for pregnant/postpartum status, age at delivery, and race. Birth outcomes were summarized by perceived stress level, combining pregnant and non-pregnant participants. For evaluating the association of perceived stress and perceived discrimination with preterm birth and SGA outcomes, unadjusted and adjusted log binomial regression models were fit to estimate the RRs and 95% CIs; for length-for-age and weight-for-length z-score outcomes, linear regression models were used to estimate the mean differences in outcomes by stress level. All analyses were performed using SAS software, Version 9.4. (SAS Institute Inc., Cary, NC, USA).

## Results

Between July 2023 and December 2024, 86 women with HIV enrolled in the SMARTT V6 study during pregnancy and 49 attended their child's age 1 year study visit and completed the online COVID-19 Impact Survey. Of these 135 surveys, 9 were excluded due to lack of response to the perceived stress question and one due to stillbirth of the respondent's infant. The remaining 125 surveys were completed by 121 unique women who were mothers to 123 enrolled infants (2 sets of twins, and 2 women who completed the survey during both pregnancy and postpartum) (see Supplementary Figure S1). Characteristics of participants by group (pregnant and postpartum) are presented in Table 1. The median age at delivery was 31.2 years [interquartile range (IQR):27.7, 35.3 years], with 57% of women self-identifying as Black/African American and 36% self-identifying as Hispanic. Overall, 24% had PHIV, with a higher percentage of PHIV women among those completing the survey during pregnancy than postpartum (27% vs. 19%). Less than 10% of women reported use of tobacco (6%), alcohol (5%), or marijuana (9%) early in pregnancy. Only 6% reported ever testing positive for COVID-19, although 42% reported that they had never tested.

**Table 1:** Characteristics of pregnant and postpartum women participating in the SMARTT maternal stress study.

Characteristic		Group^a^		
		Pregnant (*N* = 78)	Postpartum (*N* = 47)	Total (*N* = 125)
Maternal characteristics				
Age at delivery	Median (IQR)	31.3 (26.3, 35.4)	31.2 (26.7, 34.9)	31.2 (26.7, 35.3)
	Missing	2	0	2
Race	Black or African American	42 (54%)	29 (62%)	71 (57%)
	White	29 (37%)	14 (30%)	43 (34%)
	More than One Race	1 (1%)	0 (0%)	1 (1%)
	Unknown	6 (8%)	4 (9%)	10 (8%)
Ethnicity	Hispanic or Latino	31 (40%)	14 (30%)	45 (36%)
	Not Hispanic or Latino	46 (60%)	33 (70%)	79 (64%)
	Unknown	1	0	1
Site region	Northeast	9 (12%)	9 (19%)	18 (14%)
	Midwest	8 (10%)	11 (23%)	19 (15%)
	South	42 (54%)	14 (30%)	56 (45%)
	West	13 (17%)	5 (11%)	18 (14%)
	Puerto Rico	6 (8%)	8 (17%)	14 (11%)
Highest education	Less than high school	11 (17%)	12 (27%)	23 (21%)
	At least high school	54 (83%)	32 (73%)	86 (79%)
	Missing/not reported	13	3	16
Household income	≤$20,000	35 (56%)	25 (57%)	60 (57%)
	>$20,000	27 (44%)	19 (43%)	46 (43%)
	Missing	16	3	19
Mode of HIV acquisition	Non-perinatally-acquired HIV	53 (73%)	38 (81%)	91 (76%)
	Perinatally-acquired HIV	20 (27%)	9 (19%)	29 (24%)
	Unknown/Missing	5	0	5
Pre-pregnancy BMI	Median (IQR)	27.2 (24.4, 34.3)	30.8 (27.2, 36.6)	29.5 (25.6, 36.1)
	Missing	33	18	51
Earliest viral load in pregnancy	≤50 copies/mL	49 (70%)	31 (69%)	80 (70%)
	>50 copies/mL	21 (30%)	14 (31%)	35 (30%)
	Missing	8	2	10
COVID-19 infection during pregnancy	Never tested	38 (49%)	14 (30%)	52 (42%)
	Tested positive at least once	4 (5%)	4 (9%)	8 (6%)
	Always tested negative	28 (36%)	21 (45%)	49 (39%)
	Not applicable	6 (8%)	6 (13%)	12 (10%)
	Rather not answer	2 (3%)	2 (4%)	4 (3%)
Social support program assistance	Did not try to get any assistance	11 (15%)	8 (18%)	19 (16%)
	Tried and/or received assistance	61 (85%)	37 (82%)	98 (84%)
	Missing	6	2	8
Substance use in 1st trimester	Tobacco	5 (8%)	1 (2%)	6 (6%)
	Alcohol	4 (6%)	1 (2%)	5 (5%)
	Marijuana	5 (8%)	5 (12%)	10 (9%)
	Any of above	8 (12%)	6 (14%)	14 (13%)
	Missing	13	5	18
Antidepressant use in 1st trimester		3 (5%)	0 (0%)	3 (3%)
*Infant Birth Characteristics* ^b^		(*N* = 74)	(*N* = 41)	(*N* = 115)
Gestational age (weeks), median (IQR)		38.9 (37.3, 39.1)	38.0 (37.1, 39.1)	38.7 (37.1, 39.1)
Preterm birth (<37 weeks gestation)		14 (18.9%)	7 (17.1%)	21 (18.3%)
Small for gestational age (SGA)		6/72 (8.3%)	12 (29.3%)	18 (15.7%)
Growth Z-scores at birth (mean, SD)				
Length-for-age z-score		−0.23 (1.03)	−0.44 (1.09)	−0.31 (1.05)
Weight-for-age z-score		−0.40 (0.77)	−0.60 (0.88)	−0.47 (0.82)
Weight-for-length z-score		−0.23 (1.08)	−0.38 (1.20)	−0.28 (1.12)

a Two women completed a survey both during pregnancy and at their 1-year postpartum visit. Only the pregnancy survey was retained for these two women in models of pandemic-related factors and adverse birth outcomes.

b Two infants were missing weight at birth, and thus SGA status and weight Z-score were not calculated. One infant was missing length-for-age z-score. Nine infants were missing weight-for-length z-score due to missing weight, length, or values out of range for z-score calculation.

Overall, 36.0% of women reported any perceived stress during pregnancy, with similar rates for pregnant and postpartum women; 17.6% reported feeling slightly stressed, 14.4% felt moderately stressed, and only 4.0% reported feeling highly stressed (Table 2). Based on the PHQ-9, 12.2% of women screened positive for moderate or severe depression (PHQ-9 score≥10); a slightly lower percentage (9.8%) screened positive for moderate or severe anxiety (GAD-7 score≥10). A higher percentage of Black than White women reported stress (38.0% vs. 30.2%), while Hispanic women reported lower stress than non-Hispanic women (28.9% vs. 39.2%); however, the prevalence of moderate or severe depression and anxiety symptoms was similar by race and ethnicity (Supplementary Tables S2 and S3). The prevalence of high perceived discrimination (EDS score > 20) was 17.5% overall, but was twice as high for White than Black women (25.0% vs. 12.3%), although similar for Hispanic and non-Hispanic women (15.4% vs. 17.5%) (Supplementary Tables S2, S3).

**Table 2:** Perceived stress and experiences of stressors during pregnancy.

Outcome	Group		
	Pregnant (*N* = 78)	Postpartum (*N* = 47)	Total (*N* = 125)
Perceived stress level during pregnancy			
No stress	51 (65.4%)	29 (61.7%)	80 (64.0%)
Slightly stressed	12 (15.4%)	10 (21.2%)	22 (17.6%)
Moderately stressed	14 (17.9%)	4 (8.5%)	18 (14.4%)
Highly stressed	1 (1.2%)	4 (8.5%)	5 (4.0%)
Indicator of any stress during pregnancy			
Not stressed	51 (65.4%)	29 (61.7%)	80 (64.0%)
At least slightly stressed	27 (34.6%)	18 (38.3%)	45 (36.0%)
Depression symptom severity (PHQ-9) (score range)			
Minimal/no depression (0–4)	58 (75.3%)	31 (67.4%)	89 (72.4%)
Mild depression (5–9)	10 (13.0%)	9 (19.6%)	19 (15.4%)
Moderate or severe depression (≥10)	9 (11.7%)	6 (13.0%)	15 (12.2%)
Missing	1	1	2
Anxiety symptom severity (GAD-7)			
Minimal or no anxiety (0–4)	57 (74.0%)	33 (71.7%)	90 (73.1%)
Mild anxiety (5–9)	13 (16.9%)	8 (17.4%)	21 (17.1%)
Moderate or severe anxiety (≥10)	7 (9.1%)	5 (10.9%)	12 (9.8%)
Missing	1	1	2
Discrimination (Everyday Discrimination Scale)			
High discrimination (EDS>20)	16.2%	19.6%	17.5%
EDS score (median, IQR)	9 (9, 13.5)	10 (9,17)	9 (9,17)
Missing	10	1	11
Experiences of stressors^a^	*N* = 73	*N* = 45	(*N*ot combined^b^)
Emotional stress	27 (37.0%)	14 (31.1%)	
Financial stress	46 (63.0%)	27 (60.0%)	
Partner-associated stress	17 (23.3%)	18 (40.0%)	
Traumatic stress	16 (12.4%)	12 (26.7%)	
Overall stress	53 (72.6%)	38 (84.4%)	

a Pregnant WLHIV reported stressors from the PRAMS-7 (13 items) while postpartum participants reported stressors from the Stressful Life Events questionnaire (23 items). See Supplementary Tables S1 and S2 for specific questions included in each survey.

b Due to use of different surveys, responses for pregnant and postpartum participants were not combined.

The most common types of stressful life events experienced by women were financial (reported by 63% of pregnant women, 60% of postpartum women). Postpartum women reported higher occurrence than pregnant women of partner-associated stress (40.0% vs. 23.3%) and traumatic stress (26.7% vs. 12.4%). Among pregnant women reporting any perceived stress, the most commonly reported individual stressors since the pandemic started in March 2020 were having a sick family member (25%), death of a someone close to them (25%), job loss (26%), inability to pay bills (34%), or a housing change (41%) (Supplementary Table S4). Among postpartum women reporting any perceived stress, the most common reported individual stressors were death of a family member (22%), job loss (20%), a new job (29%), partner break up (22%), or more arguments than usual with partner (22%) (Supplementary Table S5).

The distribution of responses to individual questions on the COVID-19 Impact Survey is summarized in Supplementary Table S6 for social distancing behaviors, Supplementary Table S7 for trauma and violence, Supplementary Table S8 for emotions and feelings (sleep and energy level), and Supplementary Table S9 for housing, income and food insecurity. Engagement in any social distancing behavior was more common for pregnant than postpartum women (42% vs. 10%), and IPV was also more often reported in pregnant than postpartum women (25% vs. 18%), although a higher percentage of postpartum women reported having no partner. The majority of participants reported no pandemic-related change in sleep (85% for both pregnant and postpartum) or energy level (85% pregnant, 87% postpartum). Less than 10% of participants reported a moderate or significant impact of the pandemic on their daily life. Adverse impact of the pandemic on income and housing was more commonly reported, with 31% of pregnant and 26% of postpartum women reporting a decline in household income, and 18% of pregnant and 13% of postpartum women reporting a change in housing. Food insecurity was reported by 9% of both pregnant and postpartum women.

The association of depression, perceived discrimination, and the pandemic-related factors described above with any stress in pregnancy is summarized in Table 3. In both unadjusted models and in models adjusting for pregnancy status, age at delivery, and race, along with other covariates specific to each factor, women who reported engaging in social distancing, who experienced IPV, screened positive for moderate to severe depression symptoms, who had poorer sleep, lower energy levels, a housing change, or reported some pandemic impact on daily life had significantly increased risk of perceived stress in pregnancy, with RRs ranging from 2 to 5. While decline in household income was the most commonly reported pandemic-related factor, no association with perceived stress was observed.

**Table 3:** Unadjusted and adjusted associations of pandemic-related factors with risk of any perceived stress.

Factors	*N*	Unadjusted			*N*	Adjusted¹		
		Risk Ratio	95% CI	*p*-value		Risk Ratio	95% CI	*p*-value
Engaged in any social distancing behavior								
No	73	(Ref)		0.004	64	(Ref)		0.004
Yes	40	1.91	(1.23,2.96)		32	2.16	(1.29,3.62)	
Experienced IPV since the pandemic started								
No	56	(Ref)		0.01	54	(Ref)		0.02
Yes	23	2.01	(1.20,3.35)		20	1.95	(1.14,3.35)	
How the pandemic changed sleep								
No change	102	(Ref)		0.01	91	(Ref)		0.05
Made sleep worse	8	2.48	(1.71,3.59)		8	2.54	(1.56,4.15)	
Made sleep better	3	0.94	(0.19,4.78)		2	1.95	(0.68,5.61)	
How the pandemic changed daily energy levels								
No change	103	(Ref)		0.01	92	(Ref)		0.02
Made energy lower	8	2.50	(1.73,3.63)		8	3.16	(1.99,5.01)	
Made energy higher	3	0.95	(0.19,4.83)		2	1.88	(0.75,4.73)	
How much the pandemic impact daily life overall								
No impact	80	(Ref)		<0.001	70	(Ref)		< 0.001
Slight impact	24	2.96	(1.81,4.86)		23	3.35	(1.85,6.08)	
Moderate to significant impact	12	3.70	(2.29,5.98)		12	5.06	(2.83,9.06)	
How household income changed since the pandemic								
No change	52	(Ref)		0.44	43	(Ref)		0.54
Less than before	36	1.27	(0.74,2.21)		34	1.26	(0.69,2.30)	
More than before	21	1.46	(0.80,2.64)		19	1.41	(0.75,2.66)	
Housing situation changed because of the pandemic								
No	95	(Ref)		<0.001	84	(Ref)		< 0.001
Yes	20	2.37	(1.61,3.51)		17	2.59	(1.71,3.93)	
Depression symptom severity (PHQ-9)								
Minimal or no depression	85	(Ref)		<0.001	68	(Ref)		0.003
Mild depression	19	1.75	(0.97,3.15)		17	1.81	(0.87,3.75)	
Moderate to severe depression	15	3.20	(2.14,4.79)		12	3.01	(1.72,5.26)	
Perceived discrimination (EDS)								
Low discrimination	94	(Ref)		0.035	77	(Ref)		0.28
High discrimination	20	1.71	(1.04,2.81)		14	1.43	(0.75,2.71)	

CI, Confidence Interval; IPV,– intimate partner violence; PHQ-9, Patient Health Questionnaire, 9 questions.

Each factor was evaluated in a separate model, unadjusted and adjusted for the covariates noted below:

Social distancing model—adjusted for region, mode of HIV acquisition, educational attainment, and earliest viral load in pregnancy;

IPV, sleep, energy, and daily life models—adjusted for mode of HIV acquisition, earliest viral load in pregnancy; Income and housing models—adjusted for highest educational attainment.

Depression model—adjusted for any substance use during first trimester, earliest viral load in pregnancy.

Discrimination model—adjusted for highest educational attainment.

All models were adjusted for pregnant/non-pregnant status, age at delivery, and race.

Birth outcomes are summarized in Table 1, and by perceived stress level in Supplementary Table S10. Of 115 infants with data available, the median gestational age was 38.7 weeks (IQR: 37.1, 39.1 weeks), with 21 (18.3%) preterm deliveries and 18 (15.9%) infants born SGA. Overall, the mean infant weight-for-age Z-score was −0.46 (SD = 0.84) and birth length Z-score was −0.32 (SD = 1.04). No associations were observed between perceived stress in pregnancy and risk of preterm birth or SGA, or with differences in mean weight-for-age or length-for-age z-scores in either unadjusted or adjusted models (Table 4). However, women reporting higher perceived discrimination had smaller infants at birth, with 0.54 lower mean length-for-age Z-score (95%CI: −1.04, −0.04) and 0.42 lower mean weight-for-age Z-score (95% CI: −0.88, 0.04) (Table 4).

**Table 4:** Associations of perceived maternal stress and discrimination during pregnancy with birth outcomes.

Associations with perceived stress								
Binary birth outcomes	Unadjusted				Adjusted^a^			
	*N*	RR	95% CI	*p*-value	*N*	RR	95% CI	*p*-value
Preterm birth								
Not stressed	72	(Ref)	—	—	62	(Ref)	—	—
Any stress	43	0.84	(0.37,1.91)	0.67	35	0.58	(0.21,1.65)	0.31
Small for gestational age								
Not stressed	70	(Ref)	—	—	60	(Ref)	—	—
Any stress	43	1.63	(0.70,3.78)	0.26	35	0.98	(0.36,2.66)	0.97

**Continuous birth outcomes**	***N***	**mean difference**	**95% CI**	***p*-value**	***N***	**mean difference**	**95% CI**	***p*-value**
Length-for-age z-score								
Not stressed	71	(Ref)	—	—	61	(Ref)	—	—
Any stress	43	−0.23	(−0.63,0.17)	0.26	35	−0.16	(−0.56,0.24)	0.43
Weight-for-age z-score								
Not stressed	70	(Ref)	—	—	60	(Ref)		
Any stress	43	−0.13	(−0.44,0.19)	0.44	35	−0.06	(−0.41,0.28)	0.72
Weight-for-length z-score								
Not stressed	66	(Ref)	—	—	56	(Ref)	—	—
Any stress	40	0.08	(−0.37,0.53)	0.73	33	0.09	(−0.39,0.58)	0.71

**Associations with perceived discrimination**								
	**Unadjusted**				**Adjusted** ^a^			
**Binary birth outcomes**	***N***	**RR**	**95% CI**	***p*-value**	***N***	**RR**	**95% CI**	***p*-value**
Preterm birth								
Low discrimination	86	(Ref)	—	—	75	(Ref)	—	—
High discrimination	18	1.02	(0.33,3.20)	0.97	14	0.73	(0.18,2.90)	0.65
Small for gestational age								
Low discrimination	85	(Ref)	—	—	74	(Ref)	—	—
High discrimination	18	1.82	(0.74,4.46)	0.19	14	2.07	(0.62,6.87)	0.23

**Continuous birth outcomes**	***N***	**mean difference**	**95% CI**	***p*-value**	***N***	**mean difference**	**95% CI**	***p*-value**
Length-for-age z-score								
Low discrimination	86	(Ref)	—	—	75	(Ref)	—	—
High discrimination	18	−0.39	(−0.89,0.10)	0.12	14	−0.54	(−1.04,−0.04)	0.03
Weight-for-age z-score								
Low discrimination	85	(Ref)	—	—	74	(Ref)	—	—
High discrimination	18	−0.41	(−0.82,−0.00)	0.05	14	−0.42	(−0.88,0.04)	0.07
Weight-for-length z-score								
Low discrimination	80	(Ref)	—	—	70	(Ref)	—	—
High discrimination	17	−0.10	(−0.55,0.35)	0.66	13	0.02	(−0.53,0.58)	0.94

a Adjusted for race, mode of HIV acquisition, household income, and tobacco/alcohol/marijuana use.

## Discussion

During the ongoing COVID-19 pandemic, 36% of women in the SMARTT cohort reported stress during pregnancy, with 18.4% reporting moderate or high stress levels. Preiss et al. (55) suggested that such pandemic-related stress could relate to feeling unprepared for the birth of their child (preparedness stress), fears of perinatal COVID-19 infection, or both (55). Among 4,451 pregnant women recruited through social media from all 50 U.S. states to participate in the COVID-19 Pregnancy Experiences (COPE) Study, Preiss et al. observed 38.4% reporting stress. However, the COPE survey was conducted very early in the COVID-19 pandemic, whereas stress among women in the SMARTT cohort was assessed 3–4 years later and remained high. This may be partially attributable to the added burden of managing HIV, stigma, and fears of both perinatal HIV and COVID-19 infection among WLHIV. In addition, almost one fourth of women experienced the death of a family member or close friend since the pandemic started, indicating a potentially high level of emotional and traumatic events.

Surprisingly, most WLHIV (85%–90%) reported little impact of the pandemic on daily life, sleep or energy levels, and less than 13% reported moderate or severe symptoms of depression or anxiety. Nonetheless, those with depression symptoms or perceived impact of the pandemic on energy, sleep, or daily life had higher risk of stress in pregnancy. Even a slight impact of the pandemic on daily life was associated with over threefold higher risk of stress, while moderate or severe impact was associated with a 5-fold higher risk. Participants screening positive for moderate or severe depression symptoms and those reporting lower energy levels related to the pandemic had approximately threefold higher risk of stress. Compared to the relatively low proportion of WLHIV reporting an impact of the pandemic on daily life, a higher proportion reported social distancing, a change in housing situation, or experiences of IPV during the pandemic, and these factors were also strongly associated with perceived stress in pregnancy. Despite the high prevalence of financial stressors, reported by over 60% of pregnant and postpartum women, these were not associated with self-reported stress in pregnancy. Thus, the largest contributors to stress in our population were depression, isolation resulting from social distancing, disruption caused by changed in housing, and IPV.

Our study did not identify any association of self-perceived stress during pregnancy and preterm birth, SGA, or lower birth weight or length z-scores. These findings differ from other studies in the general population which have observed associations between stress and adverse pregnancy outcomes (1–5). Stress may affect preterm birth through neuroinflammatory, immune, or neuroendocrine pathways (2). For example, women with higher stress scores have previously been shown to have higher serum concentrations of IL-2 and TNF-α, and decreased concentrations of IL-10 and the vitamin D metabolite 25(OH)D (56). In a literature review conducted prior to the COVID-19 pandemic, Shapiro et al. noted that associations between stress and preterm birth tended to be strongest when stress levels were measured early in pregnancy (2), while our study evaluated stress later in pregnancy or just after delivery. Janevic et al. reported that stress, as measured by higher unemployment rates during the COVID pandemic, was associated with higher risk of preterm birth but not of SGA (57). In addition, our findings are consistent with a number of moderate to large studies conducted in Canada and Australia which observed no association between stress in pregnant women and birth outcomes during the COVID-19 pandemic (24, 58, 59). The low prevalence of self-reported anxiety in our population, one of the key drivers of stress-related effects on adverse birth outcomes, may also explain the lack of associations (2).

Given persistent racial and ethnic disparities in adverse birth outcomes and in stress levels, the role of structural racism and discrimination remains critical (60). While prior research showed that higher prevalence of stressful life events experienced by Black women contributed to such disparities based on PRAMS data, this did not fully explain higher risk of preterm birth and low birth weight observed among Black women in the U.S. (41, 42). A study of 352 women in the CARDIA study found substantially higher rates of self-reported discrimination in Black women than White women, and associations with preterm birth were substantially attenuated when adjusted for racial discrimination (61). Although Black women in our cohort reported higher rates of perceived stress during pregnancy than White women, we unexpectedly observed a higher prevalence of discrimination reported by White women than Black women. The high level of HIV-related stigma that WLHIV continue to face may contribute to the difference in our findings compared to other cohorts. Additionally, smaller sample sizes within racial subgroups led to greater uncertainty in prevalence estimates. However, among pregnant women who answered follow-up questions regarding what they perceived as the reason for discrimination, the most common response was due to their education or income level (64%), and only 36% reported their race or physical appearance to be the basis of the reported perception of discrimination.

There are several important implications of the high levels of pandemic-related stress observed among WLHIV in our study. Several studies investigating the impact of the COVID-19 pandemic on the health of pregnant women identified an important role of physical activity and of social support, and Filippetti et al. found that higher social support was associated with lower anxiety levels (27, 28). Health care via telemedicine was accelerated during the pandemic, and a number of approaches specifically targeting pregnant women and particularly those with mental health conditions have been described (62–64). A multimodal prenatal healthcare model combining in office and telemedicine visits performed favorably compared to in-office only, and remote mental health assessments were demonstrated to be feasible (62). Recognizing that digital health tools can lead to reduced healthcare engagement if not designed with trust-building and ongoing contact, Colliva proposed “10 Gold Rules for Remote Medicine Support to Pregnant Women in Critical Situations”, which prioritize mental health support, promote family involvement and other social network support, and include consistent and culturally sensitive communication (63).

We acknowledge some limitations of our study. While the COVID-19 pandemic continued to persist during our study's time frame, less virulent strains were circulating than earlier in the pandemic, and vaccination and treatment options were more widely available (65). In addition, shelter-in-place orders had been lifted, and required home schooling in some states had ceased. Although our online survey was developed in 2021, it could not be administered until other modifications to the parent protocol (SMARTT V6) were approved by our IRB, which did not occur until 2023. At the same time, while pregnant women in our study may have experienced pandemic-related stress differently than those pregnant during the height of the pandemic (2020–2022), the persistent effects of the pandemic continued to contribute to economic strain, anxiety, and other mental health issues. In addition, it resulted in reduced participation rates in this study, as experienced by most observational health research studies during this period; consequently, power for detecting associations with adverse birth outcomes was reduced.

Despite these limitations, this study has several notable strengths. It is one of the few to characterize perceived levels of stress, mental health, and discrimination among WLHIV during the COVID-19 pandemic, and to examine associations with adverse birth outcomes. We captured a wide range of possible pandemic-related factors, such as changes in housing, income, sleep, and energy levels, and associations with perceived stress during pregnancy. While we observed no association of stress during pregnancy with adverse birth outcomes, we nevertheless identified high levels of both perceived stress and experienced stressors among WLHIV. The findings of this study could inform the development of risk stratification tools to identify pregnant and postpartum WLHIV most likely to experience stress, and thus contribute to improved health outcomes in both WLHIV and their children. An important remaining knowledge gap lies in identifying and overcoming structural and institutional barriers to implementing effective and equitable approaches to delivering perinatal HIV and mental health care during large-scale public health emergencies. While the COVID-19 pandemic accelerated the use of telehealth, limited access to reliable internet service or video-capable devices likely exacerbated healthcare disparities for pregnant and postpartum WLHIV. Future studies should evaluate optimal strategies for screening maternal stress and mental health conditions and providing appropriate treatment and support during public health emergencies.

## Data Availability

The datasets presented in this study can be found in online repositories. The names of the repository/repositories and accession number(s) can be found below: NICHD Data and Specimen Hub (DASH) https://dash.nichd.nih.gov/study/227210.

## References

[1] Lilliecreutz C Laren J Sydsjo G Josefsson A. Effect of maternal stress during pregnancy on the risk for preterm birth. BMC Pregnancy Childbirth. (2016) 16:5. 10.1186/s12884-015-0775-x 26772181 PMC4714539

[2] Shapiro GD Fraser WD Frasch MG Seguin JR. Psychosocial stress in pregnancy and preterm birth: associations and mechanisms. J Perinat Med. (2013) 41(6):631–45. 10.1515/jpm-2012-0295 24216160 PMC5179252

[3] Lima SAM El Dib RP Rodrigues MRK Ferraz GAR Molina AC Neto CAP. Is the risk of low birth weight or preterm labor greater when maternal stress is experienced during pregnancy? A systematic review and meta-analysis of cohort studies. PLoS One. ( 2018) 13(7):e0200594. 10.1371/journal.pone.0200594 30048456 PMC6061976

[4] Alson JG Robinson WR Pittman L Doll KM. Incorporating measures of structural racism into population studies of reproductive health in the United States: a narrative review. Health Equity. (2021) 5(1):49–58. 10.1089/heq.2020.0081 33681689 PMC7929921

[5] McCloskey L Bernstein J, The Bridging the Chasm Collaborative LSD, Amutah-Onukagha N Anthony J Barger M Belanoff C. Bridging the chasm between pregnancy and health over the life course: a national agenda for research and action. Womens Health Issues. (2021) 31(3):204–18. 10.1016/j.whi.2021.01.002 33707142 PMC8154664

[6] Slomian J Honvo G Emonts P Reginster JY Bruyere O. Consequences of maternal postpartum depression: a systematic review of maternal and infant outcomes. Womens Health (Lond). (2019) 15:1745506519844044. 10.1177/1745506519844044 31035856 PMC6492376

[7] Oyetunji A Chandra P. Postpartum stress and infant outcome: a review of current literature. Psychiatry Res. (2020) 284:112769. 10.1016/j.psychres.2020.112769 31962260

[8] Neiger R. Long-term effects of pregnancy complications on maternal health: a review. J Clin Med. (2017) 6(8):76. 10.3390/jcm6080076 28749442 PMC5575578

[9] Pariente G Kessous R Sergienko R Sheiner E. Is preterm delivery an independent risk factor for long-term maternal kidney disease? J Matern Fetal Neonatal Med. (2017) 30(9):1102–7. 10.1080/14767058.2016.1205022 27334094

[10] Almasi O Pariente G Kessous R Sergienko R Sheiner E. Association between delivery of small-for-gestational-age neonate and long-term maternal chronic kidney disease. J Matern Fetal Neonatal Med. (2016) 29(17):2861–286. 10.3109/14767058.2015.1107896 26593268

[11] Menon R. Preterm birth: a global burden on maternal and child health. Pathog Glob Health. (2012) 106(3):139–40. 10.1179/204777312X13462106637729 23265368 PMC4001570

[12] Strauss RS. Adult functional outcome of those born small for gestational age: twenty-six-year follow-up of the 1970 British birth cohort. JAMA. (2000) 283(5):625–32. 10.1001/jama.283.5.625 10665702

[13] Barker DJ. The developmental origins of insulin resistance. Horm Res. (2005) 64(Suppl 3):2–7. 10.1159/000089311 16439838

[14] Barker DJ. The fetal origins of adult hypertension. J Hypertens Suppl. (1992) 10(7):S39–44. 10.1097/00004872-199212000-00004 1291655

[15] Barker DJ. Fetal origins of coronary heart disease. Br Heart J. (1993) 69(3):195–6. 10.1136/hrt.69.3.195 8461215 PMC1024978

[16] Lampe MA Nesheim SR Oladapo KL Ewing AC Wiener J Kourtis AP. Achieving elimination of perinatal HIV in the United States. Pediatrics. (2023) 151:e2022059604. 10.1542/peds.2022-059604 37070379 PMC10387171

[17] Perzow SED Hennessey EP Hoffman MC Grote NK Davis EP Hankin BL. Mental health of pregnant and postpartum women in response to the COVID-19 pandemic. J Affect Disord Rep. (2021) 4:100123. 33649750 10.1016/j.jadr.2021.100123 PMC7904453

[18] Feeding America. The Impact of Coronavirus on Food Insecurity (2020). Available online at: https://www.feedingamericaaction.org/the-impact-of-coronavirus-on-food-insecurity/ (Accessed March 17, 2025).

[19] Brookings. COVID-19 job and income loss leading to more hunger and financial hardship (2021). Available online at: https://www.brookings.edu/blog/up-front/2020/07/13/covid-19-job-and-income-loss-leading-to-more-hunger-and-financial-hardship/ (Accessed March 17, 2021).

[20] Center on Budget and Policy Priorities. Tracking the COVID-19 Recesssion’s Effects on Food, Housing, and Employment Hardships (2021). Available online at: https://www.cbpp.org/research/poverty-and-inequality/tracking-the-covid-19-recessions-effects-on-food-housing-and (Accessed March 17, 2021).

[21] Nicola M Alsafi Z Sohrabi C Kerwan A Al-Jabir A Iosifidis C. The socio-economic implications of the coronavirus pandemic (COVID-19): a review. Int J Surg. (2020) 78:185–93. 10.1016/j.ijsu.2020.04.018 32305533 PMC7162753

[22] Zheng J Morstead T Sin N Klaiber P Umberson D Kamble S. Psychological distress in North America during COVID-19: the role of pandemic-related stressors. Soc Sci Med. (2021) 270:113687. 10.1016/j.socscimed.2021.113687 33465600 PMC9757831

[23] Panchal N Saunders H Rudowitz R; Cox C for the Kaiser Family Foundation. The Implications of COVID-19 for Mental Health and Substance Use (2023). Available online at: https://www.kff.org/coronavirus-covid-19/issue-brief/the-implications-of-covid-19-for-mental-health-and-substance-use/ (Accessed November 18, 2025).

[24] Pearson J Fréchette-Boilard G Baudry C Matte-Gagné C Bernier A Lemelin JP. Prenatal maternal stress during the COVID-19 pandemic and birth outcomes: is the newborn spared? Infant Behav Dev. (2023) 72:101866. 10.1016/j.infbeh.2023.101866 37506422

[25] Twanow JE McCabe C Ream MA. The COVID-19 pandemic and pregnancy: impact on mothers and newborns. Semin Pediatr Neurol. (2022) 42:100977. 10.1016/j.spen.2022.100977 35868726 PMC9122838

[26] Reid CN Salinas-Miranda A Vamos C Fryer Segro K Beckstead J Sappenfield WM. Pregnant women’s experiences of stress during the COVID-19 pandemic: a qualitative study. Healthcare (Basel). (2025) 14(1):14. 10.3390/healthcare14010014 41516945 PMC12786056

[27] Dorvil S Jimenez R Pierre J Valdez J Arbelaez S Shiman LJ. The emotional and social impact of COVID-19 on postpartum women: a qualitative analysis on the experiences of women who gave birth during the pandemic in New York city. Womens Health (Lond). (2025) 21:17455057251398252. 10.1177/17455057251398252 41310930 PMC12660643

[28] Filippetti ML Clarke ADF Rigato S. The mental health crisis of expectant women in the UK: effects of the COVID-19 pandemic on prenatal mental health, antenatal attachment and social support. BMC Pregnancy Childbirth. (2022) 22(1):68. 10.1186/s12884-022-04387-7 35081906 PMC8790719

[29] Davenport MH Meyer S Meah VL Strynadka MC Khurana R. Moms are not OK: COVID-19 and maternal mental health. Front Glob Womens Health. (2020) 1:1. 10.3389/fgwh.2020.00001 34816146 PMC8593957

[30] Ho-Fung C Andersson E Hsuan-Ying H Acharya G Schwank S. Self-reported mental health status of pregnant women in Sweden during the COVID-19 pandemic: a cross-sectional survey. BMC Pregnancy Childbirth. (2022) 22(1):260. 10.1186/s12884-022-04553-x 35351030 PMC8960205

[31] Wisniewski K Henry N Flanagan AY Popoola A Weaver N Iglio L. Examining the impact of the syndemic on black birthing individuals in the USA: a systematic review. J Racial Ethn Health Disparities. (2026) 13(2):1013–27. 10.1007/s40615-025-02311-1 39994154 PMC12930410

[32] Alcindor ML. Healthcare inequities in the underserved population beyond the fanfare surrounding a pandemic: COVID-19. Evid Based Nurs. (2023) 26(2):61. 10.1136/ebnurs-2022-103572 36788009

[33] Carrasco Z Behimino A Jiles M Taylor B Clary C Negrete G. COVID-19 Impact on black and Latina women: pregnancy and parenting. J Racial Ethn Health Disparities. (2026) 13(1):540–51. 10.1007/s40615-024-02266-9 39804503 PMC12795946

[34] Chinn J Sedighim S Kirby KA Hohmann S Hameed AB Jolley J. Characteristics and outcomes of women with COVID-19 giving birth at US academic centers during the COVID-19 pandemic. JAMA Netw Open. (2021) 4(8):e2120456. 10.1001/jamanetworkopen.2021.20456 34379123 PMC8358731

[35] Congdon JL Kair LR Flaherman VJ Wood KE LoFrumento MA Nwaobasi-Iwuh E. Management and early outcomes of neonates born to women with SARS-CoV-2 in 16 U.S. Hospitals. Am J Perinatol. (2021) 38(6):622–31. 10.1055/s-0041-1726036 33723834 PMC8191701

[36] Flaherman VJ Afshar Y Boscardin WJ Keller RL H Mardy A Prahl MK. Infant outcomes following maternal infection with severe acute respiratory syndrome coronavirus 2 (SARS-CoV-2): first report from the pregnancy coronavirus outcomes registry (PRIORITY) study. Clin Infect Dis. (2021) 73(9):e2810–3. 10.1093/cid/ciaa1411 32947612 PMC7543372

[37] Lopez L 3rd Hart LH 3rd Katz MH. Racial and ethnic health disparities related to COVID-19. JAMA. (2021) 325(8):719–20. 10.1001/jama.2020.26443 33480972

[38] Gelaye B Foster S Bhasin M Tawakol A Fricchione G. SARS-CoV-2 morbidity and mortality in racial/ethnic minority populations: a window into the stress related inflammatory basis of health disparities? Brain Behav Immun Health. (2020) 9:100158. 10.1016/j.bbih.2020.100158 33052326 PMC7543984

[39] Gravlee CC. Systemic racism, chronic health inequities, and COVID-19: a syndemic in the making? Am J Hum Biol. (2020) 32(5):e23482. 10.1002/ajhb.23482 32754945 PMC7441277

[40] Krouse HJ. COVID-19 and the widening gap in health inequality. Otolaryngol Head Neck Surg. (2020) 163(1):65–6. 10.1177/0194599820926463 32366172

[41] Lu MC Chen B. Racial and ethnic disparities in preterm birth: the role of stressful life events. Am J Obstet Gynecol. (2004) 191(3):691–9. 10.1016/j.ajog.2004.04.018 15467527

[42] Almeida J Becares L Erbetta K Bettegowda VR Ahluwalia IB. Racial/ethnic inequities in low birth weight and preterm birth: the role of multiple forms of stress. Matern Child Health J. (2018) 22(8):1154–63. 10.1007/s10995-018-2500-7 29442278 PMC10998226

[43] Van Dyke RB Chadwick EG Hazra R Williams PL Seage GR 3rd; The PHACS SMARTT Study. Assessment of the safety of in utero exposure to antiretroviral drugs. Front Immunol. (2016) 7:199. 10.3389/fimmu.2016.00199 27242802 PMC4876360

[44] Williams PL Seage GR Van Dyke RB Siberry GK Griner R Tassiopoulos K. A trigger-based design for evaluating the safety of in utero antiretroviral exposure in uninfected children of human immunodeficiency virus-infected mothers. Am J Epidemiol. (2012) 175(9):950–61. 10.1093/aje/kwr401 22491086 PMC3390009

[45] Williams PL Hazra R Van Dyke RB Yildirim C Crain MJ Seage GR. Antiretroviral exposure during pregnancy and adverse outcomes in HIV-exposed uninfected infants and children using a trigger-based design. AIDS. (2016) 30(1):133–44. 10.1097/QAD.0000000000000916 26731758 PMC4704129

[46] Spitzer RL Kroenke K Williams JB Lowe B. A brief measure for assessing generalized anxiety disorder: the GAD-7. Arch Intern Med. (2006) 166(10):1092–7. 10.1001/archinte.166.10.1092 16717171

[47] Kroenke K Spitzer RL Williams JB. The PHQ-9: validity of a brief depression severity measure. J Gen Intern Med. (2001) 16(9):606–13. 10.1046/j.1525-1497.2001.016009606.x 11556941 PMC1495268

[48] Kuczmarski RJ Ogden CL Guo SS Grummer-Strawn LM Flegal KM Mei Z. 2000 CDC Growth Charts for the United States: methods and development. Vital Health Stat 11. (2002) (246):1–190.PMID: 12043359 12043359

[49] Stanhope KK Hogue CJ. Stressful life events among new mothers in Georgia: variation by race, ethnicity and nativity. Matern Child Health J. (2020) 24(4):447–55. 10.1007/s10995-020-02886-7 31993934

[50] Centers for Disease Control and Prevention. PRAMS (2020). Available online at: https://www.cdc.gov/PRAMS/ (Accessed August 3, 2020).

[51] Shulman HB D'Angelo DV Harrison L Smith RA Warner L. The pregnancy risk assessment monitoring system (PRAMS): overview of design and methodology. Am J Public Health. (2018) 108(10):1305–13. 10.2105/AJPH.2018.304563 30138070 PMC6137777

[52] Butjosa A Gómez-Benito J Myin-Germeys I Barajas A Baños I Usall J. Development and validation of the questionnaire of stressful life events (QSLE). J Psychiatr Res. (2017) 95:213–23. 10.1016/j.jpsychires.2017.08.016 28886449

[53] Williams DR Yan Y Jackson JS Anderson NB. Racial differences in physical and mental health: socio-economic Status, stress and discrimination. J Health Psychol. (1997) 2(3):335–51. 10.1177/135910539700200305 22013026

[54] Hendershot T Pan H Haines J Harlan WR Marazita ML McCarty CA. Using the PhenX toolkit to add standard measures to a study. Curr Protoc Hum Genet. (2015) 86:1.21.1–17. 10.1002/0471142905.hg0121s86 26132000 PMC8029591

[55] Preis H Mahaffey B Heiselman C Lobel M. Vulnerability and resilience to pandemic-related stress among U.S. women pregnant at the start of the COVID-19 pandemic. Soc Sci Med. (2020) 266:113348. 10.1016/j.socscimed.2020.113348 32927382 PMC7474815

[56] McLeod C Ebeling MD Baatz JE Shary JR Mulligan JR Wagner CL. Sociodemographic factors affecting perceived stress during pregnancy and the association with immune-mediator concentrations. J Perinat Med. (2021) 50(2):192–9. 10.1515/jpm-2021-0227 34757701

[57] Janevic T Lieb W Ibroci E Lynch J Lieber M Molenaar NM. The influence of structural racism, pandemic stress, and SARS-CoV-2 infection during pregnancy with adverse birth outcomes. Am J Obstet Gynecol MFM. (2022) 4(4):100649. 10.1016/j.ajogmf.2022.100649 35462058 PMC9022447

[58] Gorgui J Tchuente V Pages N Fareh T King S Elgbeili G. The impact of prenatal maternal mental health during the COVID-19 pandemic on birth outcomes: two nested case-control studies within the CONCEPTION cohort. Can J Public Health. (2023) 114(5):755–73. 10.17269/s41997-023-00814-0 37668893 PMC10485209

[59] Gladstone ME Paquin V McLean MA Lequertier B Elgbeili G Kildea S. Prenatal maternal stress was not associated with birthweight or gestational age at birth during COVID-19 restrictions in Australia: the BITTOC longitudinal cohort study. Aust N Z J Obstet Gynaecol. (2023) 63(4):509–15. 10.1111/ajo.13673 37029926

[60] Mendez DD Hogan VK Culhane JF. Institutional racism, neighborhood factors, stress, and preterm birth. Ethnicity Health. (2014) 19(5):479–99. 10.1080/13557858.2013.846300 24134165 PMC11219028

[61] Mustillo S Krieger N Gunderson EP Sidney S McCreath H Kiefe CI. Self-reported experiences of racial discrimination and black-white differences in preterm and low-birthweight deliveries: the CARDIA study. Am J Public Health. (2004) 94(12):2125–31. 10.2105/AJPH.94.12.2125 15569964 PMC1448602

[62] Ferrara A Greenberg M Zhu Y Avalos LA Ngo A Shan J. Prenatal health care outcomes before and during the COVID-19 pandemic among pregnant individuals and their newborns in an integrated US health system. JAMA Netw Open. (2023) 6(7):e2324011. 10.1001/jamanetworkopen.2023.24011 37462973 PMC10354684

[63] Colliva C Rivi V Sarti P Ferretti A Ganassi G Aguzzoli L. A pilot study on a reliable and accessible approach to remote mental health assessment: lessons from Italian pregnant women during the COVID-19 pandemic. Healthcare (Basel). (2025) 13(21):2762. 10.3390/healthcare13212762 41228129 PMC12610608

[64] Lara-Cinisomo S Ramirez Olarte A Rosales M Barrera AZ. A systematic review of technology-based prevention and treatment interventions for perinatal depression and anxiety in Latina and African American women. Matern Child Health J. (2021) 25(2):268–81. 10.1007/s10995-020-03028-9 33389589

[65] Banga J Jackson-Gibson M Diseko M Caniglia EC Mayondi G Mabuta J. No impact of COVID-19 at delivery on maternal mortality or infant adverse birth outcomes in Botswana during thr omicron era. PLoS One. (2024) 19(9):e0310980. 10.1371/journal.pone.0310980 39321175 PMC11423995

