# Supplementary material for: Maternal stress in pregnancy and adverse birth outcomes among women living with HIV during the COVID-19 pandemic
Source: Front Reprod Health. 2026 Aug 25;8:1900562. doi: 10.3389/frph.2026.1900562 (PMC13550161; doi:10.3389/frph.2026.1900562)
Supplement: Supplementary file 1 [file Datasheet1.pdf]

Supplemental Digital Content 1. Supplemental Tables and Figures

Supplemental Figure 1. Selection of study population for maternal stress study

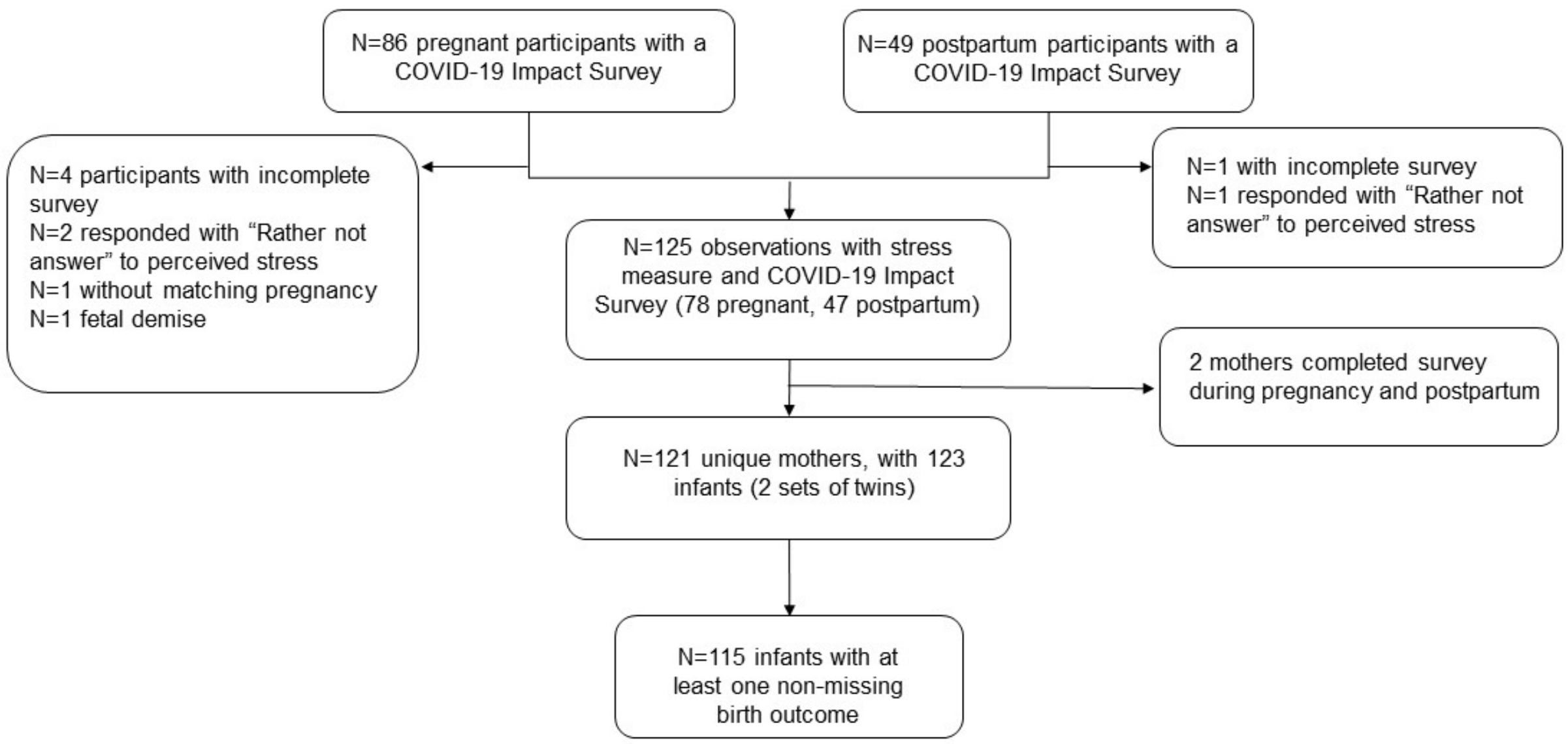

Supplemental Table 1. Stressful Life Events (SLE) questions from the PRAMS-7 for pregnant women and from the Stress and Discrimination survey for postpartum women, by domain

Domain	SLE questions asked of pregnant participants (13 items from PRAMS-7)	SLE questions asked of postpartum participants (23 items)
Emotional	• A close family member was very sick and had to go into the hospital<br>• Someone very close to you died	• You were in the hospital or were very sick or injured<br>• A family member was in the hospital or was very sick or injured<br>• A close friend died<br>• A family member or partner died
Financial	• You moved to a new address<br>• Your husband or partner lost his job<br>• You had a lot of bills you could not pay<br>• You lost your job even though you wanted to go on working	• You had to move to a new home<br>• You started a new job or got a promotion<br>• You began school or entered a new school or college<br>• You lost a job<br>• You lost financial help that you were receiving such as food stamps or Temporary Assistance for Needy Families (TANF)<br>• You lost your health insurance or access to medical care or treatment
Partner-associated	• You got separated or divorced from your husband or partner<br>• You argued with your husband or partner more than usual<br>• Your husband or partner said he did not want you to be pregnant	• You had a new boyfriend, girlfriend, or partner<br>• You got married<br>• You had a break-up with your boyfriend, girlfriend, or partner<br>• You separated or divorced from your wife, husband, or partner<br>• You had more arguments than usual with a family member or someone close to you
Traumatic	• You were homeless<br>• You were in a physical fight<br>• You or your husband or partner went to jail<br>• Someone very close to you had a bad problem with drinking or drugs	• You were homeless<br>• You had to sleep outside, in a car, or in a homeless shelter<br>• You had legal issues or interactions with the legal system<br>• You were incarcerated or spent time overnight in jail or prison<br>• A family member of someone close to you had a drug or alcohol problem<br>• You witnessed violence or heard a fight, in which a weapon was used<br>• You were physically attacked<br>• You saw or heard a violent argument involving people you care about

Supplemental Table 2. Stress, discrimination, and mental health measures by self-reported race

Characteristic		Self-reported race			
		White (N=43)	Black (N=71)	Other/ Unknown[1] (N=11)	Total (N=125)
Perceived stress level during pregnancy (ordinal)	Not stressed	30 (69.8%)	44 (62.0%)	6 (54.5%)	80 (64.0%)
	Slightly stressed	5 (11.6%)	15 (21.1%)	2 (18.2%)	22 (17.6%)
	Moderately or highly stressed	8 (18.6%)	12 (16.9%)	3 (27.3%)	23 (18.4%)
Perceived stress level during pregnancy (dichotomized - any stress)	Not stressed	30 (69.8%)	44 (62.0%)	6 (54.5%)	80 (64.0%)
	At least slightly stressed	13 (30.2%)	27 (38.0%)	5 (45.5%)	45 (36.0%)
Depression severity (PHQ-9)	Minimal or no depression	30 (69.8%)	53 (76.8%)	6 (54.5%)	89 (72.4%)
	Mild depression	9 (20.9%)	9 (13.0%)	1 (9.1%)	19 (15.4%)
	Moderate depression	2 (4.7%)	6 (8.7%)	4 (36.4%)	12 (9.8%)
	Moderately severe depression	1 (2.3%)	0 (0.0%)	0 (0.0%)	1 (0.8%)
	Severe depression	1 (2.3%)	1 (1.4%)	0 (0.0%)	2 (1.6%)
	Missing	0	2	0	2
Moderate/severe depression symptoms (PHQ9>=10)	No	39 (90.7%)	62 (89.9%)	7 (63.6%)	108 (87.8%)
	Yes	4 (9.3%)	7 (10.1%)	4 (36.4%)	15 (12.2%)
	Missing	0	2	0	2
Anxiety severity (GAD-7)	Minimal or no anxiety	25 (58.1%)	57 (82.6%)	8 (72.7%)	90 (73.2%)
	Mild anxiety	13 (30.2%)	6 (8.7%)	2 (18.2%)	21 (17.1%)
	Moderate anxiety	1 (2.3%)	5 (7.2%)	1 (9.1%)	7 (5.7%)
	Severe anxiety	4 (9.3%)	1 (1.4%)	0 (0.0%)	5 (4.1%)
	Missing	0	2	0	2
Moderate/severe anxiety symptoms (GAD7>=10)	No	38 (88.4%)	63 (91.3%)	10 (90.9%)	111 (90.2%)
	Yes	5 (11.6%)	6 (8.7%)	1 (9.1%)	12 (9.8%)
	Missing	0	2	0	2
Everyday discrimination scale (EDS) score[2]	Median (IQR)	9.00 (9.0, 19.6)	9 (9, 14)	11 (9, 11)	9 (9, 17)
High perceived discrimination (EDS score >20)	No	30 (75.0%)	57 (87.7%)	7 (77.8%)	94 (82.5%)
	Yes	10 (25.0%)	8 (12.3%)	2 (22.2%)	20 (17.5%)
	Missing	3	6	2	11

IQR=interquartile range (25th, 75th percentiles)

[1]-The "Other/Unknown race" included 1 reporting more than one race, and 10 who chose not to report race.
[2]-The EDS score is the sum of 9 questions about experiences of discrimination, each with 6 levels from 1 (never) to 6 (every day); the total score has a range of 9 (never to all 9 questions) to 54 (every day to all 9 questions)

Supplemental Table 3. Stress, discrimination, and mental health measures by ethnicity

Characteristic		Ethnicity: Hispanic or Latino (N=45)	Not Hispanic or Latino (N=79)	Unknown (N=1)	Total (N=125)
Perceived stress level during pregnancy (ordinal)	Not stressed	32 (71.1%)	48 (60.8%)	0 (0.0%)	80 (64.0%)
	Slightly stressed	3 (6.7%)	18 (22.8%)	1 (100.0%)	22 (17.6%)
	Moderately or highly stressed	10 (22.2%)	13 (16.5%)	0 (0.0%)	23 (18.4%)
Perceived stress level during pregnancy (dichotomized - any stress)	Not stressed	32 (71.1%)	48 (60.8%)	0 (0.0%)	80 (64.0%)
	At least slightly stressed	13 (28.9%)	31 (39.2%)	1 (100.0%)	45 (36.0%)
Depression severity (PHQ-9)	Minimal or no depression	30 (66.7%)	59 (76.6%)	0 (0.0%)	89 (72.4%)
	Mild depression	9 (20.0%)	10 (13.0%)	0 (0.0%)	19 (15.4%)
	Moderate depression	5 (11.1%)	6 (7.8%)	1 (100.0%)	12 (9.8%)
	Moderately severe depression	0 (0.0%)	1 (1.3%)	0 (0.0%)	1 (0.8%)
	Severe depression	1 (2.2%)	1 (1.3%)	0 (0.0%)	2 (1.6%)
	Missing	0	2	0	2
Moderate/severe depression symptoms (PHQ9>=10)	No	39 (86.7%)	69 (89.6%)	0 (0.0%)	108 (87.8%)
	Yes	6 (13.3%)	8 (10.4%)	1 (100.0%)	15 (12.2%)
	Missing	0	2	0	2
Anxiety severity (GAD-7)	Minimal or no anxiety	31 (68.9%)	59 (76.6%)	0 (0.0%)	90 (73.2%)
	Mild anxiety	10 (22.2%)	11 (14.3%)	0 (0.0%)	21 (17.1%)
	Moderate anxiety	1 (2.2%)	5 (6.5%)	1 (100.0%)	7 (5.7%)
	Severe anxiety	3 (6.7%)	2 (2.6%)	0 (0.0%)	5 (4.1%)
	Missing	0	2	0	2
Moderate/severe anxiety symptoms (GAD7>=10)	No	41 (91.1%)	70 (90.9%)	0 (0.0%)	111 (90.2%)
	Yes	4 (8.9%)	7 (9.1%)	1 (100.0%)	12 (9.8%)
	Missing	0	2	0	2
Everyday discrimination scale (EDS) score[1]	Median (IQR)	9 (9, 15)	9 (9, 17)	32 (32, 32)	9 (9, 17)
High perceived discrimination (EDS score >20)	No	33 (84.6%)	61 (82.4%)	0 (0.0%)	94 (82.5%)
	Yes	6 (15.4%)	13 (17.6%)	1 (100.0%)	20 (17.5%)
	Missing	6	5	0	11

IQR=interquartile range (25th, 75th percentiles)

[1]-The EDS score is the sum of 9 questions about experiences of discrimination, each with 6 levels from 1 (never) to 6 (every day); the total score has a range of 9 (never to all 9 questions) to 54 (every day to all 9 questions)

Supplemental Table 4. Responses to individual stressful life events (stressors) from the CDC PRAMS-7 questionnaire by reported perceived stress level for pregnant participants

Stressors		Perceived stress			
		Not stressed (N=48)	Slightly stressed (N=10)	Moderately or highly stressed (N=15)	Total (N=73)
**Emotional stressors**					
Family member was sick?	Yes	10 (21%)	1 (10%)	7 (47%)	18 (25%)
	No	38 (79%)	9 (90%)	8 (53%)	55 (75%)
Someone very close to you died?	Yes	8 (17%)	2 (20%)	8 (53%)	18 (25%)
	No	40 (83%)	8 (80%)	7 (47%)	55 (75%)
**Financial stressors**					
Moved to a new address?	Yes	19 (40%)	3 (30%)	8 (53%)	30 (41%)
	No	29 (60%)	7 (70%)	7 (47%)	43 (59%)
Husband or partner lost his job?	Yes	9 (19%)	2 (20%)	3 (20%)	14 (19%)
	No	39 (81%)	8 (80%)	12 (80%)	59 (81%)
Had a lot of bills you could not pay?	Yes	14 (29%)	5 (50%)	6 (40%)	25 (34%)
	No	34 (71%)	5 (50%)	9 (60%)	48 (66%)
Lost a job?	Yes	10 (21%)	6 (60%)	3 (20%)	19 (26%)
	No	38 (79%)	4 (40%)	12 (80%)	54 (74%)
**Partner-associated stressors**					
Separated from partner?	Yes	5 (10%)	0 (0%)	3 (20%)	8 (11%)
	No	43 (90%)	10 (100%)	12 (80%)	65 (89%)
Had more arguments than usual?	Yes	4 (8%)	3 (30%)	3 (20%)	10 (14%)
	No	44 (92%)	7 (70%)	12 (80%)	63 (86%)
Husband or partner said he did not want you to be pregnant?	Yes	3 (6%)	0 (0%)	1 (7%)	4 (5%)
	No	45 (94%)	10 (100%)	14 (93%)	69 (95%)
**Traumatic stressors**					
Were homeless?	Yes	4 (8%)	1 (10%)	3 (20%)	8 (11%)
	No	44 (92%)	9 (90%)	12 (80%)	65 (89%)
Were in a physical fight?	Yes	1 (2%)	0 (0%)	1 (7%)	2 (3%)
	No	47 (98%)	10 (100%)	14 (93%)	71 (97%)
You or your partner went to jail?	Yes	1 (2%)	0 (0%)	2 (13%)	3 (4%)
	No	47 (98%)	10 (100%)	13 (87%)	70 (96%)
Someone very close to you had a drug problem?	Yes	3 (6%)	2 (20%)	3 (20%)	8 (11%)
	No	44 (94%)	8 (80%)	12 (80%)	64 (89%)
	Missing	1	0	0	1

Supplemental Table 5. Responses to individual stressful life events (stressors) by reported perceived stress level for postpartum participants

Stressors		Perceived stress: Not stressed (N=28)	Perceived stress: Slightly stressed (N=9)	Perceived stress: Moderately or highly stressed (N=8)	Total (N=45)
**Emotional stressors**					
Sick or in the hospital?	Yes	2 (7%)	0 (0%)	0 (0%)	2 (4%)
	No	26 (93%)	9 (100%)	8 (100%)	43 (96%)
Family member was sick?	Yes	1 (4%)	2 (22%)	3 (38%)	6 (13%)
	No	27 (96%)	7 (78%)	5 (63%)	39 (87%)
Family member or partner died?	Yes	5 (18%)	2 (22%)	3 (38%)	10 (22%)
	No	23 (82%)	7 (78%)	5 (63%)	35 (78%)
Close friend died?	Yes	3 (11%)	1 (11%)	2 (25%)	6 (13%)
	No	25 (89%)	8 (89%)	6 (75%)	39 (87%)
**Financial stressors**					
Moved to a new home?	Yes	7 (25%)	1 (11%)	2 (25%)	10 (22%)
	No	21 (75%)	8 (89%)	6 (75%)	35 (78%)
Started a new job or got a promotion?	Yes	9 (32%)	2 (22%)	2 (25%)	13 (29%)
	No	19 (68%)	7 (78%)	6 (75%)	32 (71%)
Began new school or college?	Yes	2 (7%)	0 (0%)	2 (25%)	4 (9%)
	No	26 (93%)	9 (100%)	6 (75%)	41 (91%)
Lost a job?	Yes	5 (18%)	3 (33%)	1 (13%)	9 (20%)
	No	23 (82%)	6 (67%)	7 (88%)	36 (80%)
Lost financial help?	Yes	5 (18%)	1 (11%)	2 (25%)	8 (18%)
	No	23 (82%)	8 (89%)	6 (75%)	37 (82%)
Lost health insurance?	Yes	1 (4%)	1 (11%)	0 (0%)	2 (4%)
	No	27 (96%)	8 (89%)	8 (100%)	43 (96%)
**Partner-associated stressors**					
Had a new partner?	Yes	4 (14%)	1 (11%)	2 (25%)	7 (16%)
	No	24 (86%)	8 (89%)	6 (75%)	38 (84%)
Got married?	Yes	2 (7%)	0 (0%)	0 (0%)	2 (4%)
	No	26 (93%)	8 (89%)	8 (100%)	42 (93%)
	Rather not answer	0 (0%)	1 (11%)	0 (0%)	1 (2%)
Broke up with partner?	Yes	6 (21%)	0 (0%)	3 (38%)	9 (20%)
	No	21 (75%)	9 (100%)	5 (63%)	35 (78%)
	Rather not answer	1 (4%)	0 (0%)	0 (0%)	1 (2%)
Separated from partner?	Yes	4 (14%)	0 (0%)	3 (38%)	7 (16%)
	No	24 (86%)	9 (100%)	5 (63%)	38 (84%)

Stressors		Perceived stress: Not stressed (N=28)	Slightly stressed (N=9)	Moderately or highly stressed (N=8)	Total (N=45)
Had more arguments than usual?	Yes	7 (25%)	1 (11%)	2 (25%)	10 (22%)
	No	21 (75%)	8 (89%)	6 (75%)	35 (78%)
**Traumatic stressors**					
Were homeless?	Yes	3 (11%)	1 (11%)	1 (13%)	5 (11%)
	No	25 (89%)	8 (89%)	7 (88%)	40 (89%)
Slept outside or in car or shelter?	Yes	2 (7%)	1 (11%)	2 (25%)	5 (11%)
	No	26 (93%)	8 (89%)	6 (75%)	40 (89%)
Had trouble with law?	Yes	1 (4%)	0 (0%)	1 (13%)	2 (4%)
	No	27 (96%)	9 (100%)	7 (88%)	43 (96%)
Incarcerated?	No	28 (100%)	9 (100%)	8 (100%)	45 (100%)
Family member had drug problem?	Yes	4 (14%)	0 (0%)	2 (25%)	6 (13%)
	No	24 (86%)	9 (100%)	6 (75%)	39 (87%)
Witness fight where weapon used?	Yes	2 (7%)	0 (0%)	0 (0%)	2 (4%)
	No	26 (93%)	9 (100%)	8 (100%)	43 (96%)
Physically attacked?	Yes	1 (4%)	0 (0%)	0 (0%)	1 (2%)
	No	27 (96%)	9 (100%)	8 (100%)	44 (98%)
Saw/heard violent argument?	Yes	2 (7%)	0 (0%)	1 (13%)	3 (7%)
	No	26 (93%)	9 (100%)	7 (88%)	42 (93%)

Supplemental Table 6. Survey responses to social distancing behavior for pregnant and postpartum women

Question		Group	
		Pregnant (N=78)	Postpartum (N=47)
Engaged in social distancing behavior	Yes	30 (42%)	10 (22%)
	No	42 (58%)	35 (78%)
	Missing	6	2
Reduced or stopped: In-person contact with family inside home	Reduced or stopped	5 (6%)	4 (9%)
	Not reduced	53 (68%)	30 (64%)
	Does not apply	14 (18%)	11 (23%)
	Rather not answer	6 (8%)	2 (4%)
Reduced or stopped: In-person contact with family who live outside home	Reduced or stopped	16 (21%)	3 (6%)
	Not reduced	45 (58%)	31 (66%)
	Does not apply	12 (15%)	11 (23%)
	Rather not answer	5 (6%)	2 (4%)
Reduced or stopped: In-person contact with friends	Reduced or stopped	12 (15%)	5 (11%)
	Not reduced	49 (63%)	29 (62%)
	Does not apply	12 (15%)	11 (23%)
	Rather not answer	5 (6%)	2 (4%)
Reduced or stopped: In-person contact with other pregnant women or parents	Reduced or stopped	10 (13%)	3 (6%)
	Not reduced	44 (56%)	24 (51%)
	Does not apply	18 (23%)	18 (38%)
	Rather not answer	6 (8%)	2 (4%)
Reduced or stopped: In-person contact at work	Reduced or stopped	10 (13%)	4 (9%)
	Not reduced	38 (49%)	28 (60%)
	Does not apply	25 (32%)	13 (28%)
	Rather not answer	5 (6%)	2 (4%)
Reduced or stopped: Regular childcare by outside care provider	Reduced or stopped	4 (5%)	2 (4%)
	Not reduced	39 (50%)	27 (57%)
	Does not apply	30 (38%)	16 (34%)
	Rather not answer	5 (6%)	2 (4%)
Reduced or stopped: Family or personal travel	Reduced or stopped	9 (12%)	5 (11%)
	Not reduced	46 (59%)	30 (64%)
	Does not apply	18 (23%)	10 (21%)
	Rather not answer	5 (6%)	2 (4%)
Reduced or stopped: Activities in outdoor spaces	Reduced or stopped	11 (14%)	5 (11%)
	Not reduced	50 (64%)	30 (64%)
	Does not apply	12 (15%)	10 (21%)
	Rather not answer	5 (6%)	2 (4%)
Reduced or stopped: Activities in public spaces	Reduced or stopped	15 (19%)	5 (11%)
	Not reduced	48 (62%)	30 (64%)
	Does not apply	10 (13%)	10 (21%)

Question		Group	
		Pregnant (N=78)	Postpartum (N=47)
	Rather not answer	5 (6%)	2 (4%)
Reduced or stopped: Going to restaurants or stores	Reduced or stopped	16 (21%)	5 (11%)
	Not reduced	48 (62%)	30 (64%)
	Does not apply	9 (12%)	10 (21%)
	Rather not answer	5 (6%)	2 (4%)
Reduced or stopped: Indoor exercise classes or recreational sports	Reduced or stopped	11 (14%)	5 (11%)
	Not reduced	40 (51%)	24 (51%)
	Does not apply	22 (28%)	16 (34%)
	Rather not answer	5 (6%)	2 (4%)
Reduced or stopped: In-person events in the community	Reduced or stopped	11 (14%)	5 (11%)
	Not reduced	42 (54%)	27 (57%)
	Does not apply	20 (26%)	13 (28%)
	Rather not answer	5 (6%)	2 (4%)
Reduced or stopped: In-person religious services	Reduced or stopped	11 (14%)	5 (11%)
	Not reduced	39 (50%)	27 (57%)
	Does not apply	22 (28%)	13 (28%)
	Rather not answer	6 (8%)	2 (4%)

Supplemental Table 7. Survey responses to trauma and violence questions on the COVID-19 Impact Survey for pregnant and postpartum women

Question		Group	
		Pregnant (N=78)	Postpartum (N=47)
Since pandemic started: How safe felt in neighborhood	Not safe at all	3 (4%)	0 (0%)
	Not very safe	3 (4%)	2 (4%)
	Somewhat safe	19 (24%)	10 (21%)
	Very safe	43 (55%)	30 (64%)
	Don't know	7 (9%)	4 (9%)
	Rather not answer	3 (4%)	1 (2%)
Since pandemic started: Level of violence in neighborhood	Increased	8 (10%)	4 (9%)
	Decreased	4 (5%)	6 (13%)
	Stayed the same	41 (53%)	24 (51%)
	Don't know	21 (27%)	12 (26%)
	Rather not answer	4 (5%)	1 (2%)
Since pandemic started: Crime rate in neighborhood	More often	6 (8%)	5 (11%)
	Less often	6 (8%)	6 (13%)
	Stayed the same	37 (47%)	25 (53%)
	Don't know	24 (31%)	10 (21%)
	Rather not answer	5 (6%)	1 (2%)
In past year: Close friend or relative die or seriously injured due to violence?	Yes	10 (13%)	2 (4%)
	No	64 (82%)	34 (72%)
	Don't know	3 (4%)	9 (19%)
	Rather not answer	1 (1%)	2 (4%)
In past year: Have you, friend or relative been robbed or attacked?	Yes	10 (13%)	2 (4%)
	No	64 (82%)	40 (85%)
	Don't know	3 (4%)	4 (9%)
	Rather not answer	1 (1%)	1 (2%)
In past year: Heard gunshots in community?	Yes	27 (35%)	17 (36%)
	No	46 (59%)	25 (53%)
	Don't know	3 (4%)	3 (6%)
	Rather not answer	2 (3%)	2 (4%)
In past year: Witnessed violence or crime in neighborhood?	Yes	7 (9%)	5 (11%)
	No	68 (87%)	38 (81%)
	Don't know	2 (3%)	2 (4%)
	Rather not answer	1 (1%)	2 (4%)
Since pandemic started: How safe felt in own home	Not safe at all	3 (4%)	1 (2%)
	Somewhat safe	13 (17%)	6 (13%)
	Very safe	57 (73%)	35 (74%)
	Don't know	1 (1%)	2 (4%)
	Rather not answer	4 (5%)	3 (6%)
How do your feelings about safety in home compare to before pandemic	I feel less safe	3 (4%)	3 (6%)

Question		Group	
		Pregnant (N=78)	Postpartum (N=47)
	I feel the same	56 (72%)	35 (74%)
	I feel safer	11 (14%)	6 (13%)
	Rather not answer	8 (10%)	3 (6%)
Since pandemic started: Partner yelled or made you feel bad?	Never	39 (67%)	21 (66%)
	Sometimes	13 (22%)	5 (16%)
	Often	3 (5%)	1 (3%)
	Rather not answer	3 (5%)	5 (16%)
	Missing	20	15
Since pandemic started: Partner push or grab you in anger?	Never	48 (83%)	26 (81%)
	Sometimes	6 (10%)	3 (9%)
	Often	1 (2%)	0 (0%)
	Rather not answer	3 (5%)	3 (9%)
	Missing	20	15
Since pandemic started: Partner force sexual contact?	Never	52 (90%)	28 (88%)
	Sometimes	3 (5%)	0 (0%)
	Rather not answer	3 (5%)	4 (13%)
	Missing	20	15
Experienced intimate partner violence since the pandemic started	Yes	16 (25%)	7 (18%)
	No	38 (59%)	21 (55%)
	Did not have partner	10 (16%)	10 (26%)
	Missing	14	9

Supplemental Table 8. Survey responses to emotions and feelings questions on the COVID-19 Impact Survey for pregnant and postpartum women

Question		Group	
		Pregnant (N=78)	Postpartum (N=47)
How has pandemic changed sleep	Made my sleep a lot worse	1 (1%)	1 (2%)
	Made my sleep a little worse	3 (4%)	3 (6%)
	No change	66 (85%)	40 (85%)
	Made my sleep a little better	1 (1%)	1 (2%)
	Made my sleep a lot better	1 (1%)	0 (0%)
	Rather not answer	6 (8%)	2 (4%)
How has pandemic changed daily energy levels	Made my energy a lot lower	1 (1%)	0 (0%)
	Made my energy somewhat lower	5 (6%)	2 (4%)
	No change	66 (85%)	41 (87%)
	Made my energy somewhat higher	0 (0%)	1 (2%)
	Made my energy a lot higher	1 (1%)	1 (2%)
	Rather not answer	5 (6%)	2 (4%)
How much has the pandemic impacted your daily life overall	No impact	53 (68%)	31 (66%)
	Slight impact	15 (19%)	9 (19%)
	Moderate impact	5 (6%)	3 (6%)
	Significant impact	2 (3%)	2 (4%)
	Rather not answer	3 (4%)	2 (4%)

Supplemental Table 9. Survey responses to housing, income and food insecurity questions on the COVID-19 Impact Survey for pregnant and postpartum women

Question		Group	
		Pregnant (N=78)	Postpartum (N=47)
Since pandemic started: Household income changed a lot?	Yes, my household income is more than before	14 (18%)	8 (17%)
	Yes, my household income is less than before	24 (31%)	12 (26%)
	No, my household income is about the same	31 (40%)	24 (51%)
	Rather not answer	9 (12%)	3 (6%)
Housing situation changed because of pandemic?	Yes	14 (18%)	6 (13%)
	No	59 (76%)	40 (85%)
	Rather not answer	5 (6%)	1 (2%)
In general, how stressed about employment and finances due to pandemic	No stress	37 (47%)	30 (64%)
	Slightly stressed	18 (23%)	7 (15%)
	Moderately stressed	10 (13%)	4 (9%)
	Highly stressed	8 (10%)	4 (9%)
	Rather not answer	5 (6%)	2 (4%)
In general, how stressed about future employment and finances due to pandemic	No stress	38 (49%)	29 (62%)
	Slightly stressed	14 (18%)	6 (13%)
	Moderately stressed	10 (13%)	5 (11%)
	Highly stressed	7 (9%)	2 (4%)
	Rather not answer	9 (12%)	5 (11%)
Stressful because of pandemic: Access to food?	No	71 (91%)	43 (91%)
	Yes	7 (9%)	4 (9%)

Supplemental Table 10: Birth outcomes by perceived stress level

Birth outcome	Perceived stress level			Total (N=121)
	Not stressed (N=76)	Slightly stressed (N=22)	Moderately or highly stressed (N=23)	
Gestational age (weeks), median (IQR)	38.9 (37.1, 39.1)	38.6 (37.3, 39.1)	38.8 (37.3, 39.3)	38.7 (37.1, 39.1)
# missing	4	1	1	6
Preterm birth (<37 weeks gestation)	14 (19.4%)	4 (19.1%)	3 (13.6%)	21 (18.3%)
Small for gestational age (SGA)	9 (12.9%)	5 (23.8%)	4 (18.2%)	18 (15.9%)
# missing	6	1	1	8
**Growth Z-scores at birth (mean, SD)***				
Length-for-age z-score	-0.22 (1.03)	-0.58 (1.26)	-0.33 (0.88)	-0.32 (1.04)
# missing	5	1	1	5
Weight-for-age z-score	-0.42 (0.77)	-0.46 (0.97)	-0.64 (0.81)	-0.46 (0.84)
# missing	6	1	1	6
Weight-for-length z-score	-0.32 (1.06)	0.07 (1.21)	-0.29 (1.21)	-0.24 (1.12)
	20	7	4	31
